# Alveolar MMP28 is associated with clinical outcomes and measures of lung injury in acute respiratory distress syndrome

**DOI:** 10.1186/s13054-020-02847-0

**Published:** 2020-04-08

**Authors:** Eric D. Morrell, Carmen Mikacenic, Ke-Qin Gong, Susanna Kosamo, Mark M. Wurfel, Anne M. Manicone

**Affiliations:** Division of Pulmonary, Critical Care, and Sleep Medicine, Center for Lung Biology, University of Washington, 850 Republican St, Seattle, WA 98109 USA

**Keywords:** Acute respiratory distress syndrome, Alveolar macrophages, Matrix metalloproteinases

Dear Editor,

Alveolar macrophages (AM) express a unique repertoire of matrix metalloproteinases (MMPs) that have downstream effects on inflammatory mediators involved in acute respiratory distress syndrome (ARDS) pathogenesis. MMP28 is the newest member of the MMP family and has been shown to be upregulated in inflammatory conditions such as idiopathic pulmonary fibrosis [1]. In animal models of lung infection, MMP28 plays a key role in macrophage chemotaxis [2] and in modulating macrophage polarity [3]. AM polarization plays a central role in orchestrating alveolar inflammation and repair in ARDS, and we have previously shown that AM transcriptional programs are associated with ARDS clinical outcomes such as ventilator-free days (VFDs) [4]. Therefore, our primary hypothesis was that AM *MMP28* gene expression is associated with VFDs in subjects with ARDS. Secondarily, we hypothesized that AM *MMP28* gene expression and alveolar MMP28 concentrations are associated with P_a_O_2_/F_i_O_2_ ratio (P/F ratio), percentage alveolar neutrophils (% PMNs), and total protein levels.

We analyzed bronchoalveolar lavage fluid (BALF) from subjects previously enrolled in a phase-II trial [5] of omega-3 fatty acids for the treatment of ARDS (*n* = 76) (Table 1). In a subset of these patients (*n* = 25), AMs were purified from BALF by negative selection as previously described [4]. Samples were obtained from subjects within 48 h of ARDS onset and prior to them receiving study drug. We extracted RNA from isolated AMs, assessed it for purity, and then reverse-transcribed it into cDNA. RT-PCR was performed per the manufacturer’s instructions using *HPRT* and *MMP28* (Hs01020031_m1) primer probe sets from Applied Biosystems. BALF MMP28 was measured using an ELISA (Cat #: LS-F12061) specific for human MMP28 per the manufacturer’s instructions (LifeSpan Biosciences). Specimens with an MMP28 concentration below the lower limit of detection (LLOD) were assigned an MMP28 concentration of 50% of the LLOD for analytical purposes. These data were analyzed with non-parametric tests. In primary analysis, we tested for associations between AM-specific *MMP28* gene expression (relative quantification) and VFDs. In secondary analysis, we tested for associations between AM-specific *MMP28* gene expression or BALF MMP28 protein levels and P/F ratio, % PMNs, and alveolar total protein levels.

**Table 1:** Subject characteristics

Characteristic	ARDS cohort (*n* = 76)
Demographic	
Age (mean ± SD)	50 ± 16
Sex (M/F)	45/31
Comorbidities	
Diabetes	16 (22%)
Cirrhosis	5 (7%)
Chronic renal insufficiency	2 (3%)
ARDS risk factor*, *n* (%)	
Sepsis	48 (64%)
Pneumonia	31 (41%)
Trauma	27 (37%)
Other	8 (11%)
Physiologic	
P/F Ratio (median, IQR)	156, (121–205)
APACHE II (mean ± SD)	22 ± 7
Outcome	
VFDs (median, IQR)	14, (0–21)
Mortality (28-day) (*n*, %)	11, 20%

*ARDS risk factors are not mutually exclusive; *APACHE* Acute Physiology, Age, Chronic Health Evaluation, *ARDS* acute respiratory distress syndrome, *IQR* interquartile range, *P/F ratio* P_a_O_2_/F_i_O_2_ ratio, *SD* standard deviation, *VFDs* ventilator-free days—defined as the number of days a subject is alive and free from mechanical ventilation between day 1 and day 28 after enrollment. If a subject died before day 28, they were considered to have VFDs = 0

Higher AM *MMP28* gene expression at the time of ARDS onset was associated with worse VFDs (Fig. 1a, groups were dichotomized by the median VFDs). We next tested whether AM *MMP28* gene expression on day 1 was associated with P/F ratio to determine whether there was a link between AM *MMP28* gene expression and a lung-specific endpoint. Higher AM *MMP28* gene expression was associated with worse P/F ratio (Fig. 1b groups were divided into mild-moderate (P/F > 150) vs. moderate-severe (P/F < 150) based on a recent classification of ARDS severity [6]). In secondary analysis, we found that higher BALF MMP28 concentrations were associated with worse P/F ratio, but not VFDs (Fig. 1c, d). Increased BALF MMP28 concentrations were associated with increased % PMNs and total protein concentrations (Fig. 1e, f).

**Fig. 1:**
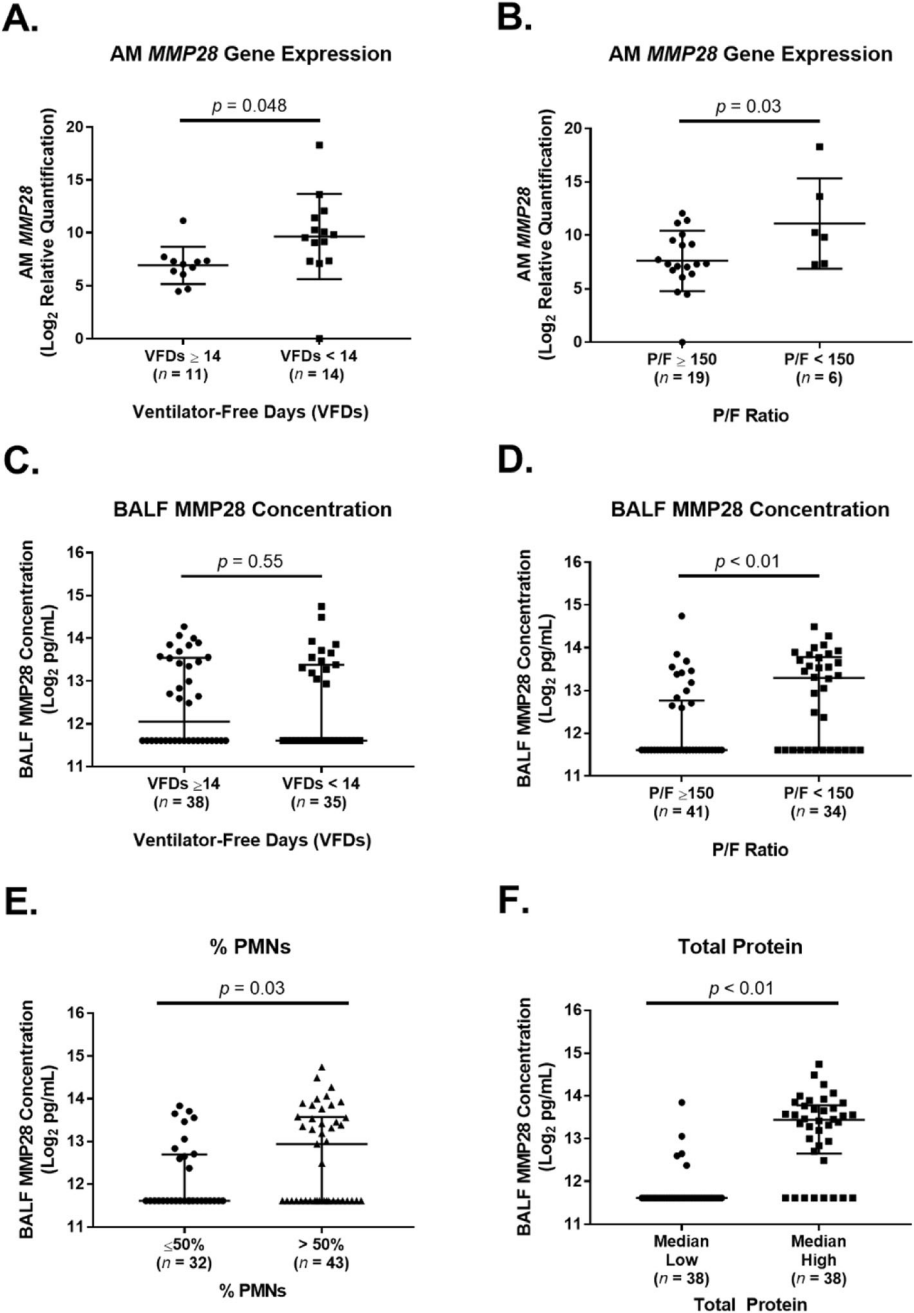
Alveolar MMP28 is associated with clinical outcomes in subjects with acute respiratory distress syndrome (ARDS). **a** AM-specific relative gene expression of *MMP28* was higher in subjects with worse ventilator-free days (VFDs) (VFDs < 14) vs. better VFDs (VFDs ≥ 14) (*p* = 0.048, unpaired *t* test). Subjects were divided by the median VFDs (VFD = 14). Shown are the individual values, mean, and standard deviation. **b** AM-specific relative gene expression of *MMP28* was higher in subjects with a P/F ratio < 150 vs. P/F ratio ≥ 150 (*p* = 0.03, unpaired *t* test). Shown are the individual values, mean, and standard deviation. C) BALF MMP28 concentrations were not different in subjects with worse VFDs (VFDs < 14) vs. better VFDs (VFDs ≥14) (*p* = 0.55, Wilcoxon rank test). Shown are the individual values, median, and interquartile range. **d** BALF MMP28 concentrations were higher in subjects with a P/F ratio < 150 vs. P/F ratio ≥ 150 (*p* < 0.01, Wilcoxon rank test). Shown are the individual values, median, and interquartile range. **e** BALF MMP28 concentrations were higher in subjects with % PMNs > 50% vs. subjects with % PMNs ≤50% (*p* = 0.03, Wilcoxon rank test). Shown are the individual values, median, and interquartile range. **f** BALF MMP28 concentrations were higher in subjects with higher alveolar total protein vs. subjects with lower alveolar total protein (*p* < 0.01, Wilcoxon rank test). Subjects were divided by the median alveolar total protein concentration (306.5 μg/mL). Shown are the individual values, median, and interquartile range

Our study is the first in humans to demonstrate that increased AM *MMP28* gene expression within the first 48 h after ARDS onset is associated with worse VFDs. Future studies that employ alveolar sampling are needed to validate the findings from this single-cohort association study.

## Data Availability

The datasets used and analyzed during the current study are available from the corresponding author on reasonable request.

## Abbreviations

AM: Alveolar macrophage
ARDS: Acute respiratory distress syndrome
BALF: Bronchoalveolar lavage fluid
ELISA: Enzyme-linked immunosorbent assay
MMP: Matrix metalloproteinases
P/F ratio: P_a_O_2_/F_i_O_2_ ratio
RQ: Relative quantification
VFD: Ventilator-free days
